# Supplementary material for: Does perceived quality mediate the relationship between country of origin image and consumer buying behaviour in Nigerian hypermarkets?
Source: PLoS One. 2023 Aug 29;18(8):e0285438. doi: 10.1371/journal.pone.0285438 (PMC10464966; doi:10.1371/journal.pone.0285438)
Supplement: Attachment — Submitted filename: Response to Reviewers_PONE.docx [file pone.0285438.s001.docx]

**Reviewer #1: I enjoyed reading the piece of research.
It is very clearly organizes, written in good English.
I have just a few suggestions that could improve the paper.
I have not particularly enjoyed the chapter regarding consumer behavior. I think more interesting and newer information could have been presented regarding this aspect.**

**The focus of this section is on conceptualizing consumer buying behaviour and the steps involved. However, it was discovered that this objective wasn’t entirely meet as it did not earlier define what consumer buying behaviour is all about. Thus, the gap has been filled with a clear definition. Aside that, the section is still in line with the objective of conceptualizing consumer buying behaviour and steps involved.**

**At the end of the article we should be able to see the limits of the research and the future research suggestions, and the implications for practitioners and researchers. The contributions of this paper should also be stated and underlined in the Conclusions chapter.**

**All these issues are very important and it was an error on our side to omit them. They have been addressed.**

**Reviewer #2: This research is good. However to make it better, please clarify the methodology parts and indicate the sampling methods and sampling process as they are ambiguous.**

**Done**

**Please revisit your references and properly write them alphabetically if possible unless it fulfilled the journal requirements.**

**It follows journal requirement / style**

**Please also re-consider the length of this writeup and be precise on your literature review.**

**The journal requires that work be concise and fully explained, irrespective of number of words. They don’t have limitations on length.**

**Your conclusion is not comprehensive enough to support the whole study and please also indicate the implication of this research practically and theoretically. If possible, please mention clearly the future research needs. Best wishes.**

**This issue has been addressed in the work**
